# A global analysis of CNVs in swine using whole genome sequence data and association analysis with fatty acid composition and growth traits

**DOI:** 10.1371/journal.pone.0177014

**Published:** 2017-05-04

**Authors:** Manuel Revilla, Anna Puig-Oliveras, Anna Castelló, Daniel Crespo-Piazuelo, Ediane Paludo, Ana I. Fernández, Maria Ballester, Josep M. Folch

**Affiliations:** 1 Departament de Ciència Animal i dels Aliments, Facultat de Veterinària, Universitat Autònoma de Barcelona (UAB), Bellaterra, Spain; 2 Plant and Animal Genomics, Centre de Recerca en Agrigenòmica (CRAG), Consorci CSIC-IRTA-UAB-UB, Campus UAB, Bellaterra, Spain; 3 Department of Animal Science, Santa Catarina State University, Lages, Santa Catarina, Brazil; 4 Departamento de Genética Animal, Instituto Nacional de Investigación y Tecnología Agraria y Alimentaria (INIA), Madrid, Spain; 5 Departament de Genètica i Millora Animal, Institut de Recerca i Tecnologia Agroalimentàries (IRTA), Torre Marimon, Caldes de Montbui, Spain; Universita degli Studi di Bologna, ITALY

## Abstract

Copy number variations (CNVs) are important genetic variants complementary to SNPs, and can be considered as biomarkers for some economically important traits in domestic animals. In the present study, a genomic analysis of porcine CNVs based on next-generation sequencing data was carried out to identify CNVs segregating in an Iberian x Landrace backcross population and study their association with fatty acid composition and growth-related traits. A total of 1,279 CNVs, including duplications and deletions, were detected, ranging from 106 to 235 CNVs across samples, with an average of 183 CNVs per sample. Moreover, we detected 540 CNV regions (CNVRs) containing 245 genes. Functional annotation suggested that these genes possess a great variety of molecular functions and may play a role in production traits in commercial breeds. Some of the identified CNVRs contained relevant functional genes (e.g., *CLCA4*, *CYP4X1*, *GPAT2*, *MOGAT2*, *PLA2G2A* and *PRKG1*, among others). The variation in copy number of four of them (*CLCA4*, *GPAT2*, *MOGAT2* and *PRKG1*) was validated in 150 BC1_LD (25% Iberian and 75% Landrace) animals by qPCR. Additionally, their contribution regarding backfat and intramuscular fatty acid composition and growth–related traits was analyzed. Statistically significant associations were obtained for CNVR112 (*GPAT2*) for the C18:2(n-6)/C18:3(n-3) ratio in backfat and carcass length, among others. Notably, GPATs are enzymes that catalyze the first step in the biosynthesis of both triglycerides and glycerophospholipids, suggesting that this CNVR may contribute to genetic variation in fatty acid composition and growth traits. These findings provide useful genomic information to facilitate the further identification of trait-related CNVRs affecting economically important traits in pigs.

## Introduction

The pig (*Sus scrofa*) is one of the most economically important livestock animals worldwide, and one of the main sources of animal meat for humans. The pig is also a valuable animal model for human diseases and nutrition. In recent years, genomic structural variations have received considerably more attention, as they represent the major source of genetic variation in mammalian genomes in terms of number of nucleotides involved [1]. Copy number variations (CNVs) are a type of genetic structural variation which corresponds to relatively large regions of the genome (typically larger than 1 kb) that have been deleted or duplicated, giving different numbers of copies of a DNA fragment [2]. CNVs can affect both gene expression and regulation, with potentially large phenotypic effects [3]. In humans, several studies on CNVs showed association with Mendelian diseases and complex genetic disorders, such as schizophrenia [4], cancer [5,6], and various congenital defects [7]. In pigs, CNVs have been associated with several phenotypes such as coat color [8], backfat (BF) thickness [3] and meat quality [9], demonstrating that CNVs can be considered as promising biomarkers for some economically important traits in domestic animals. Fat content and fatty acid (FA) composition determine important sensory and technological aspects of pork and meat products because of their influence on the melting point and oxidative status of porcine tissues [10]. Artificial selection to increase meat production in pigs has caused a reduction of intramuscular fat (IMF) and changes in meat FA composition in some breeds. Pork quality is important to the meat-processing industry, therefore a higher IMF content and a better FA profile, while maintaining a reduced amount of BF, is a main selection objective [11,12].

In the past few years, different approaches have been used to detect CNVs in pig genomes: array comparative genomic hybridization (aCGH) [13,14], high-density single nucleotide polymorphisms genotyping [3, 15–19] and next-generation sequencing (NGS) of whole genomes [20–23]. Ramayo-Caldas *et al*. [15] reported the first whole genome description of CNVs in the pig genome using genotypes from the 60K SNP chip (*Illumina*). Fernández *et al*. [24] also applied the SNP array method on 217 highly inbred Iberian pigs, and then used high-throughput sequencing on four of those pigs for validation. Bickhart *et al*. [20] demonstrated that the NGS has superiority over the SNP chip and aCGH in CNV detection in livestock genomes. The aCGH and SNP arrays have been extensively used for CNV screens, however, these techniques are often affected by low probe density and cross-hybridization of repetitive sequences [20]. The influence and utilization of NGS and complementary analysis programs have provided better approaches to detect CNVs at the genome-wide level [25].

The goal of this study is to identify CNV regions (CNVRs) from whole genome sequence (WGS) data on autosomal chromosomes, using an Iberian x Landrace (IBMAP) cross, validate a selection of them in a larger number of animals and study their association with growth and meat quality traits.

## Materials and methods

### Ethics statement

All animal procedures were performed according to the Spanish Policy for Animal Protection RD1201/05, which meets the European Union Directive 86/609 concerning the protection of animals used in experimentation. Animals were sacrificed in a commercial slaughterhouse following national and institutional guidelines for the Good Experimental Practices and approved by the Ethical Committee of the Institution (IRTA- Institut de Recerca i Tecnologia Agroalimentàries).

### Animal samples

Seven founders of the IBMAP experimental population [26], two Iberian boars (Guadyerbas line) and five Landrace sows, were used to identify CNVs by NGS of whole genomes. Furthermore, thirty-two individuals of different backcrosses: BC1_LD (25% Iberian and 75% Landrace; n = 10), BC1_PI (25% Iberian and 75% Pietrain; n = 10), and BC1_DU (25% Iberian and 75% Duroc; n = 12) were used to test the variability of six computationally-predicted CNVs. Finally, association analyses between CNVs and FA composition and growth traits were performed in 150 BC1_LD individuals from the IBMAP population. All animals were maintained under intensive conditions and feeding was *ad libitum* with a cereal-based commercial diet. Backcross animals were slaughtered at an average age of 179.8±2.6 days, and samples of diaphragm tissue were collected, snap-frozen in liquid nitrogen and stored at -80°C until analysis. Genomic DNA was extracted from diaphragm tissue samples of all animals by the phenol-chloroform method [27].

### NGS data

The whole genomes of seven founders of the IBMAP population (two Iberian boars and five Landrace sows) were sequenced with the Illumina Hi-Seq 2000 platform (Illumina; San Diego, CA, USA) in the CNAG institution (Centro Nacional de Análisis Genómico; Barcelona, Spain), obtaining 100 bp paired-end reads. The reads were mapped using Burrows-Wheeler Aligner software [28] to the reference genome (*Sscrofa10*.*2*), having a mean coverage of 13.1x. Sequencing information is provided in the Results section.

### Detection of CNV

Control-FREEC software [29,30] was used to detect CNVs in the genomes of all individuals. This software uses GC-content to normalize read counts, and lower mappability regions can be excluded from the analysis using provided mappability tracks [29,30]. The mapped paired-end reads files were used to calculate read count in non-overlapping windows by a sliding-window approach. An optimal windows size was selected by the program for each sample (Table 1). Then, normalization of read counts was performed by GC content in the same set of windows. At the end, the software analyzed the prediction regions for gains and losses in order to assign copy numbers to these regions. The program was run using the default parameters without any control sample. Following the recommendations of Derrien *et al*. [31] to limit the number of false positives, we used a GEM mappability file created by the GEM (Genome Multi-tool) mappability program. Then, p-values to the predicted CNVs were added by running the “rtrackplayer” R package [32], which adds both Wilcoxon test and Kolmogorov-Smirnov test p-values to each Control-FREEC prediction.

**Table 1:** Summary of CNVs of the seven analyzed pigs.

Sample	Breed	No. of total calls	No. of duplications	No. of deletions	Window size for CNV detection (kb)	Median size and (range), in kb
Ib1	Iberian	235	117	118	3.39	3.39 (3.39–1,008.02)
Ib2	Iberian	189	101	88	3.34	3.34 (3.34–2,237.31)
Ld1	Landrace	106	76	30	3.39	6.78 (3.39–416.85)
Ld2	Landrace	203	117	86	3.42	3.42 (3.42–855.75)
Ld3	Landrace	172	104	68	3.22	3.22 (3.22–366.62)
Ld4	Landrace	208	122	86	3.64	3.64 (3.64–563.58)
Ld5	Landrace	166	101	65	3.24	3.24 (3.24–447.67)
**On average**		**183**	**106**	**77**	**3.38**	**3.42 (3.22–2,237.31)**

The CNVRs were determined by merging CNVs identified in two or more animals when the overlap is of at least 1 bp, according to the criteria proposed by Redon *et al*. [1]. This merging was performed by CNVRuler software [33]. Regions of very low density of overlapping (recurrence parameter<0.1) were not used in the analyses for a more robust definition of the beginning and end regions.

### Gene content and functional annotation

Based on the *Sscrofa10*.*2* sequence assembly, pig gene annotations within the identified CNVRs were retrieved from the Ensembl Genes 84 Database using the BioMart tool of Ensembl (http://ensembl.org/biomart). Pathway analysis of these genes was performed with DAVID bioinformatics resources 6.7 (http://david.abcc.ncifcrf.gov/). Considering the limited number of genes annotated in the pig genome, we first converted the pig Ensembl gene IDs to homologous human Ensembl gene IDs by BioMart, and then carried out the pathway analysis. The *P* value and Benjamini correction for multiple testing were assessed for statistical significance.

### Real-time quantitative PCR

Real-time quantitative PCR (qPCR) was used to analyze CNVRs. Thirty-two individuals of different backcrosses: BC1_LD (n = 10), BC1_PI (n = 10) and BC1_DU (n = 12) were used to validate CNVRs. Furthermore, 150 individuals of the BC1_LD were used to perform the association analysis between CNVRs and FA composition and growth traits. The 2^-ΔΔCt^ method [34] for relative quantification (RQ) of CNVRs was used as previously described in Ramayo-Caldas *et al*. [15].

Primers (S1 Table) were designed using the Primer Express 2.0 software (Applied Biosystems). qPCRs were carried out using SYBR^®^ Select Master Mix in an ABI PRISM^®^ 7900HT instrument for primer testing (Applied Biosystems, Inc.; Foster City, CA) and a QuantStudio™ 12K Flex Real-Time PCR System (Applied Biosystems, Inc.; Foster City, CA) for the CNV quantification, following the manufacturer’s guidelines. The reactions were carried out in a 96-well plate for the ABI PRISM^®^ 7900HT instrument in a 20μl volume containing 10 ng of genomic DNA. For the QuantStudio™ 12K Flex Real-Time PCR instrument, the reactions were carried out in a 384-well plate in 15μl volume containing 7.5 ng of genomic DNA. All primers were used at 300 nM. The thermal cycle was: 10 min at 95°C, 40 cycles of 15 sec at 95°C and 1 min at 60°C. Each sample was analyzed in triplicate. One sample without CNV for each of the genomic regions analyzed was used as reference. The control region was determined within the region of the glucagon gene [EMBL:GCG]. Results for the standard curve were analyzed by DAG Expression software [35] and all samples were analyzed with Thermo Fisher Cloud software 1.0 (Applied Biosystems). For each CNVR to be validated, a value from the 2x2^-ΔΔCt^ formula was calculated for each individual.

### Traits analyzed

For this study, phenotypic records were used from 150 animals belonging to the IBMAP BC1_LD backcross. The composition of 15 FA of both *Longissimus dorsi* muscle and BF (taken between the third and the fourth ribs) tissues was determined by gas chromatography as described in Pérez-Enciso *et al*. [26]. Subsequently, the percentage of each FA relative to the total FA was calculated as well as the global percentages of saturated fatty acids (SFA), monounsaturated fatty acids (MUFA), polyunsaturated fatty acids (PUFA) and related indices, including desaturation and elongation indices.

In addition, 16 phenotypic growth and carcass traits were used in the analysis, corresponding to body weight measured at 125, 155 and 180 days (BW125, BW155, and BW180, respectively), backfat thickness (BFT) at the level of the fourth rib at 4 cm of the midline measured by ultrasounds at 125, 155 and 180 days (BFT125, BFT155 and BFT180, respectively) and measured with a ruler at slaughter (BFT), carcass length (CRCL) and carcass weight (CW), ham weight (HW), shoulder weight (SW), belly weight (BLW) and the IMF percentage, which was measured in the *Longissimus dorsi* muscle by Near Infrared Transmittance (NIT; Infratec 1625, Tecator Hoganas). Additionally pH was measured at 45 min in *semimembranosus* muscle (pH45SM) and at 24 h (pH24LD) and 45 min in the *Longissimus dorsi* muscle (pH45LD).

### Statistical analysis

Associations of RQ values of the CNVRs with phenotypic records were analyzed with a multiplicative effect model in the CNVassoc R package [36]. The CNVassoc function incorporates calls by using a latent class model as described in González *et al*. [37]. Association analyses were performed with the copy number status inferred with the CNV function of the CNVassoc R package. The qPCR data and the composition of FA in IMF and BF were normalized and corrected both by gender and batch (five levels) effects, and the composition of FA also for CW, using glm R package [38]. Different corrections were used for the analysis of phenotypic growth records. Carcass weight was corrected by gender, batch and slaughter age. Also, gender, batch and CW were used to correct pH45SM, pH45LD, pH24LD, CRCL, BLW, BFT, HW, SW and IMF. For BFT125, BFT155 and BFT180, the corrections used were gender, batch and the body weight at their respective days. Meanwhile, for the body weight, the corrections used were gender, batch and the animal age. The R package q-value [39] was used to calculate the false-discovery rate (FDR), and the cut-off of the significant association was set at the q-value ≤ 0.05.

## Results and discussion

### Genome-wide detection of CNVs

Based on the Illumina platform (Hi-Seq 2000, Illumina; San Diego, CA, USA), WGS data of seven founders of the IBMAP population (two Iberian boars and five Landrace sows) were obtained. These animals were selected because they were founders with a large progeny contribution to the IBMAP population. The sequences were 100 bp paired-end reads with a coverage per animal ranging from 12.1 to 13.8x, with an average of 13.1x, which is sufficient for genome-wide CNV detection using the Read Depth method according to previous studies [20].

A total number of 1,279 CNVs, after removing false positives, were predicted from all seven individuals in autosomal chromosomes. The number of CNV events ranged from 106 to 235 CNVs across samples, with an average of 183 CNVs per sample. The size of these CNVs ranged from 3.22 to 2,237.31 kb per sample, with a median size of 3.42 kb (Table 1). The minimum CNV size is limited by the window size selected by the Control-FREEC program and, hence, the minimum size value includes all the CNVs with smaller sizes. The CNV median size is equal to the minimum size in six of the seven analyzed animals, indicating that most of the CNVs have sizes smaller or equal to the minimum sizes. When comparing the frequency of CNVs, duplications showed a higher average frequency than did deletions (106 versus 77). This proportion may be related to natural selection, as it is assumed that the genome is more tolerant of duplications than of deletions [40]. The overall profile of these CNVs across the genome for each individual is detailed in S2 Table.All detected CNV segments were further merged into 540 unique CNVRs (S3 Table) across all experimental animal genomes following the criteria that the union of overlapping CNVs across individuals is considered as a CNVR [1].

**Fig 1:**
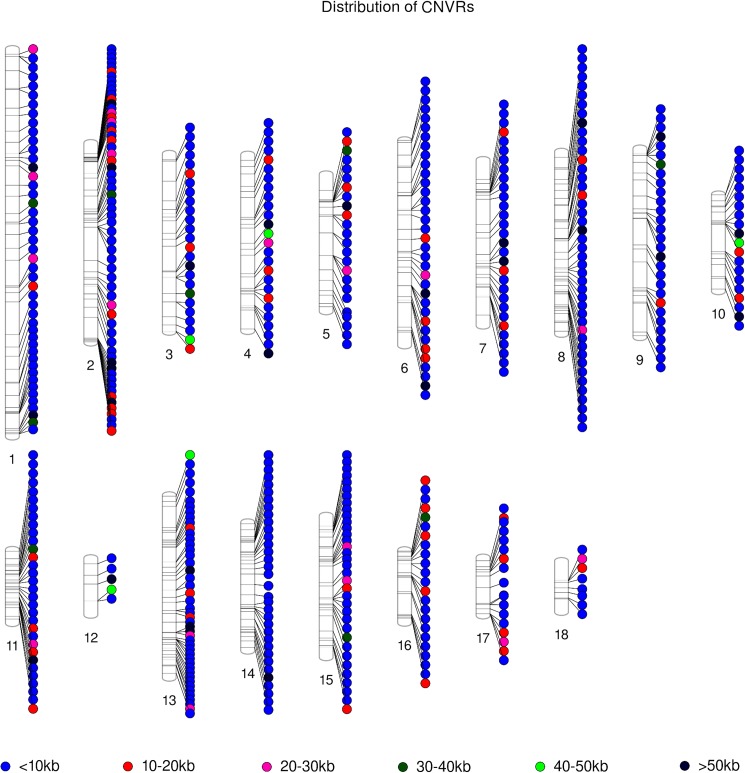
Distribution of CNVRs on the different chromosomes of the porcine genome. Each CNVR is represented by a circle and colors correspond to the different sizes explained in the legend.

Although CNVRs were found on all chromosomes, the number and the total size of CNVRs per chromosome were not correlated with chromosome length. The majority (428 out of 540; 79.26%) of the CNVRs identified were smaller than 10 kb (Fig 1).

### Comparison with CNVRs identified in previous reports

The quality of CNVR calls was assessed by a comparison against a previously reported porcine CNV dataset identified in the IBMAP population with the Porcine SNP60 BeadChip [15]. After remapping the position of the CNVRs identified in Ramayo-Caldas *et al*. [15] to the *Sscrofa10*.*2* (http://www.ncbi.nlm.nih.gov/dbvar/studies/nstd44/#varianttab), we found 32 CNVRs that overlapped with CNVRs in that dataset, accounting for 65% of their CNVR calls. Another comparison was performed against the work published by Fernández *et al*. [24] using 223 Guadyerbas Iberian pigs and based on whole genome SNP genotyping data, obtaining seven CNVRs that overlapped (S3 Table).

Potential reasons for the differences between our results and these studies may be: (i) there was a difference in population size and genetic background between our study and others; (ii) call algorithms to detect CNVs are different, and (iii) our results were based on the *Sscrofa10*.*2* genome assembly, while previous works were based on *Sscrofa* 9.0. This discrepancy between works also occurred in CNV studies of other mammals [41–43].

### Gene annotation and functional analysis of the CNVRs

In total, 245 genes within or partially inside of the identified CNVRs were retrieved from the Ensembl Genes 84 Database using the BioMart data management system, including 227 protein-coding genes, 13 pseudogenes, 2 miRNA, 1 miscRNA and 2 snRNA (S3 Table).

In order to provide insight into the functional enrichment of the CNVRs, pathway analysis was performed with the DAVID bioinformatics resource. The pathway analysis revealed that genes in CNVRs mainly participated in olfactory transduction, retinol metabolism and also in metabolism of xenobiotics by cytochrome P450 and arachidonic acid metabolism, among others (S4 Table). Olfactory transduction was the most overrepresented pathway, including 48 genes, which is consistent with the study of Paudel *et al*. [23]. These authors suggested that inter-specific CNV of olfactory receptors (ORs) facilitated rapid adaptation to different environments during the diversification of the genus *Sus*. The genes involved in retinol and arachidonic acid metabolism pathways are components of the cytochrome P450 superfamily of enzymes, which catalyzes a high variety of chemical reactions mainly involved in detoxification and hormone and lipid metabolism [44]. Together with ORs, CNV in cytochrome P450 (*CYP450*) genes suggests a relevant role of these genes in the organism’s adaptation to rapid changes in the environment [23].

Interestingly, one gene of this family: Cytochrome P4502 C32 Fragment gene (*CYP2C32*; ENSSSCG00000010488), was identified in a previous study using BC1_LD animals of the IBMAP population [15], suggesting a possible role of this structural variation in determining androsterone levels. Differential expression of genes of the *CYP450* family has been correlated with androsterone levels in pigs from Duroc and Landrace breeds [45].

In addition, other genes related to this family were identified: *CYP1A1* (ENSSSCG00000001906), *CYP19A1* (ENSSSCG00000030168), *CYP2B6* (ENSSSCG00000003006), *CYP4A24* (ENSSSCG00000024778), and *CYP4X1* (ENSSSCG00000024129), which could affect arachidonic acid metabolism. In this sense, Ramayo-Caldas *et al*. [46] demonstrated some members of this family differentially-expressed in the liver transcriptome of pigs with extreme phenotypes for intramuscular FA composition.

Also, the excess of CNVRs in intergenic regions implies that a major part of these variations are likely to be neutral [47].

### Identification of candidate genes for growth and FA composition traits in CNVRs

The overlapping was analyzed between the CNVRs identified in this study and the Genome-Wide Association Study (GWAS) regions described in Ramayo-Caldas *et al*. [48] using BC1_LD animals of the IBMAP population. After remapping these regions using the Assembly Converter tool of Ensembl, 19 regions co-localized among these works (S3 Table). The overlapping between the CNVRs and QTLs for growth and body composition traits described in Fernández *et al*. [49], which use a genome QTL scan based on linkage-mapping analyses with three generations of the IBMAP population, was also analyzed, identifying five co-localized regions (S3 Table). Finally, Muñoz *et al*. [50] also performed an analysis of the genetic basis of the FA composition of BF and IMF in the IBMAP population to identify significant QTLs for these traits using linkage-mapping and GWAS methods. A total of 24 overlapping regions were identified between both studies (S3 Table). In addition, we found 10 genes located in CNVRs that have been reported as differentially-expressed in the liver [46], muscle [51] or adipose tissue [52] of BC1_LD animals phenotypically extreme for intramuscular FA composition (S3 Table).

After compiling this information and taking into consideration the functional analysis of the genes within or partially inside of these CNVRs, six genes were selected as potential candidate genes related to growth and FA composition traits (Table 2).

**Table 2:** Selected CNVRs for validation and association studies.

CNVR ID	Chr	CNVR Start	CNVR End	Length (bp)	CNVR gene	Gene Start	Gene End	Gene region	Function	Overlapping with previous reports		
										CNVs	GWAS	DE studies^a^
112	3	48,486,060	48,496,968	10,908	*GPAT2*	48,487,404	48,497,055	All gene	Esterifies acyl-group from acyl-ACP to the sn-1 position of glycerol-3-phosphate, an essential step in glycerolipid biosynthesis			
157	4	141,944,876	142,127,882	183,006	*CLCA4*	142,080,450	142,110,266	All gene	Mediates calcium-activated chloride conductance	[15]	[48]	
198	6	72,514,368	72,520,800	6,432	*PLA2G2A*	72,517,360	72,520,303	All gene	Catalyzes the hydrolysis of the sn-2 fatty acid acyl ester bond of phosphoglycerides, releasing free fatty acids and lysophospholipids			
214	6	151,988,436	151,995,708	7,272	C*YP4X1*	151,954,326	152,007,678	I6-7/E7/I7-8/E8/I8-9	Catalyzes many reactions involved in synthesis of cholesterol, steroids and other lipids			[46]
298	9	11,128,173	11,133,432	5,259	*MOGAT2*	11,119,062	11,132,962	I2-3/E3/I3-4/E4/I4-5/E5/I5-6/E6	Absorption of dietary fat in the small intestine			[46, 52]
447	14	106,486,107	106,491,408	5,301	*PRKG1*	106,253,479	106,707,670	I2-3	Serine/threonine protein kinase that acts as key mediator of the nitric oxide (NO)/cGMP signaling			[51]

^a^Differentially-expressed genes analysis using RNA-Seq data in IBMAP animals.

These six CNVRs represent different predicted statues of copy numbers (duplication/deletion) and are located on different chromosomes (SSC3, SSC4, SSC6, SSC9 and SSC14):

1. CNVR112 contains the *GPAT2* gene (ENSSSCG00000008121), encoding the mitochondrial glycerol-3-phosphate acyl-transferase 2, which plays a key role in phospholipid and triacylglycerol biosynthesis by catalyzing the addition of fatty acylCoA at the sn1 position of glycerol-3-phosphate to form lyso-phosphatidic acid [53]. Among its related pathways are metabolism and regulation of lipid metabolism by Peroxisome proliferator-activated receptor alpha *(PPARA)*.
2. The *CLCA4* gene (ENSSSCG00000006932), located in CNVR157, may be involved in mediating calcium-activated chloride conductance [54]. The porcine *CLCA4* gene has recently been shown to be duplicated into two separated genes, *CLCA4a* and *CLCA4b* [55].
3. CNVR198 contains the *PLA2G2A* gene (ENSSSCG00000003494), which encodes an enzyme that catalyzes the hydrolysis of the sn-2 FA acyl-ester bond of phosphoglycerides, releasing FAs and lysophospholipids, and could participate in the regulation of the phospholipid metabolism in biomembranes [56].
4. The *CYP4X1* gene (ENSSSCG00000024129), identified inside CNVR214, encodes a member belonging to the cytochrome P450 superfamily of enzymes. As stated before, the cytochrome P450 proteins are monooxygenases which catalyze many reactions involved in drug metabolism and synthesis of cholesterol, steroids and other lipids [44].
5. The *MOGAT2* gene (ENSSSCG00000014861), found in CNVR298, encodes a monoacylglycerol O-acyltransferase 2 enzyme. It plays a central role in absorption of dietary fat in the small intestine by catalyzing the re-synthesis of digested triacylglycerol in enterocytes. This gene may contribute to the development of the fatty-pig phenotype [57].
6. The *PRKG1* gene (ENSSSCG00000010429), located in CNVR447, has been implicated in the nitric oxide signaling pathway [58], one of the most significantly over-represented pathways found in the muscle RNA-Seq analysis of differentially-expressed genes for FA composition traits [51].

### Validation of CNVRs

**Fig 2:**
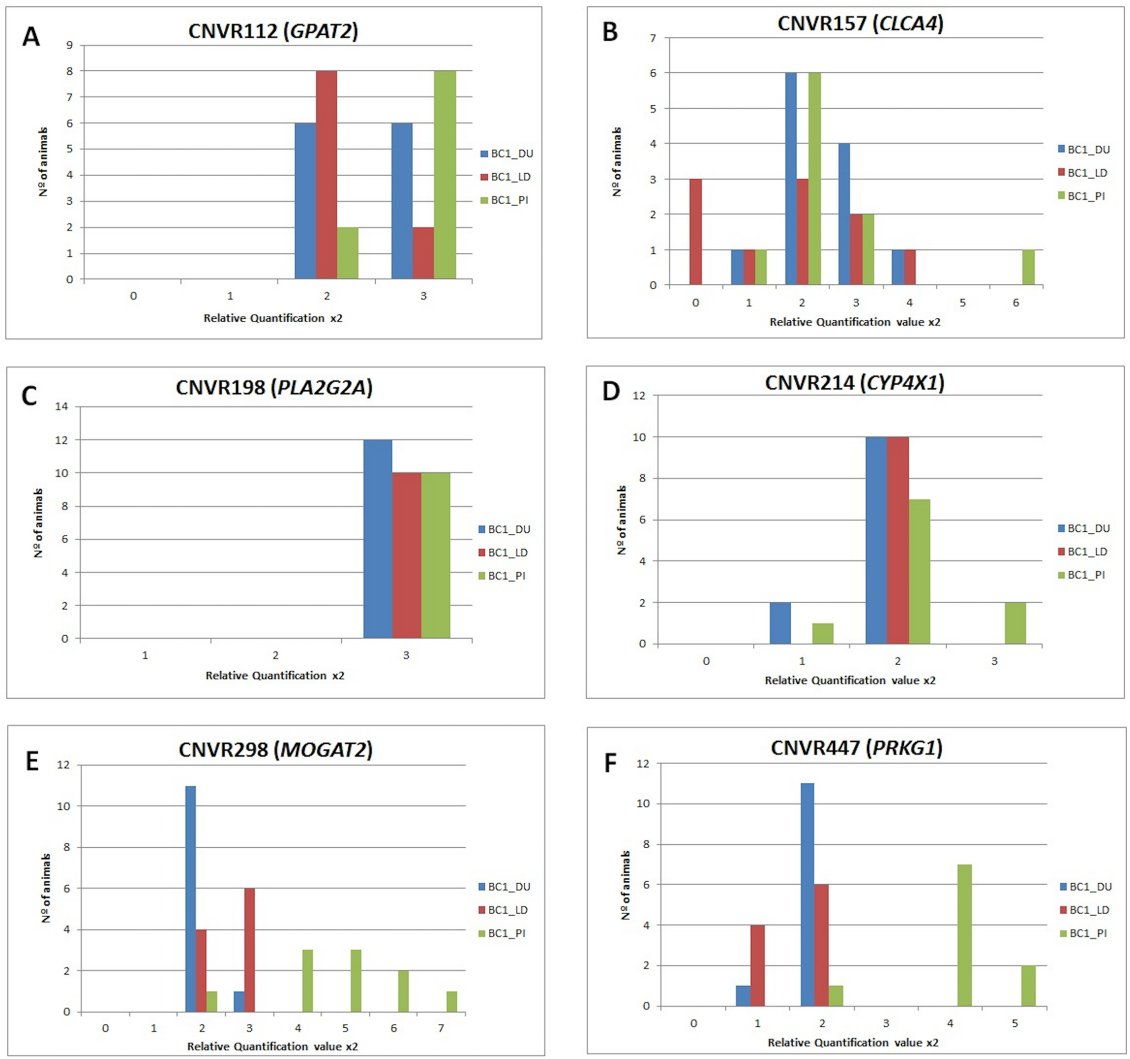
Validation of CNVRs detected from the WGS data using qPCR analysis. The y-axis represents the animals and the x-axis shows the relative quantification value (2^-ΔΔCt^ values for qPCR; 2*(2^Sample signal) values for qPCR).

In order to validate the six selected CNVRs (CNVRs 112, 157, 198, 214, 298, and 447; Table 2), qPCR assays were designed. We analyzed the variation of these CNVRs in 12, 10 and 10 animals belonging to BC1_DU, BC1_LD and BC1_PI backcrosses, respectively. CNV was observed among these animals for five of the six analyzed CNVRs (112, 157, 214, 298, and 447), showing different patterns of CNV among the backcrosses (Fig 2). For CNVR112 (*GPAT2*), animals with two and three copies were observed in the three backcrosses. CNVR157 (*CLCA4*) showed the highest variability in the three backcrosses, with a CNV ranging from 0 to 6 copies among individuals from the different backcrosses. Conversely, for CNVR214 (*CYP4X1*), no variation in copy number was observed in BC1_LD animals, and it was discarded for further analyses. CNVR298 (*MOGAT2*) and CNVR447 (*PRKG1*) also showed variation in the number of copies among animals of the three backcrosses, in both cases being the individuals of the BC1_PI which presented more variation, as compared with the other two backcrosses.

### Association analysis with growth, carcass and fatty acid composition traits

With the objective to carry out association analysis between the CNVRs and growth-related and meat quality traits, 150 animals of BC1_LD were tested for CNVRs 112, 157, 298, and 447 (S1–S4 Figs). The repeatability of qPCR triplicates was highly accurate, showing a maximum standard error of 0.03. Results for CNVR157 (*CLCA4*) revealed that copy number varied greatly among the BC1_LD population (S2 Fig). The distribution of RQ values for CNVR112 (*GPAT2*) and CNVR298 (*MOGAT2*) also revealed variability and the differences between the calibrator and the sample that presented the highest value of RQ was 0.78 for CNVR112 and 0.77 for CNVR298 (S1 and S3 Figs). CNVR447 (*PRKG1*) variation was more homogeneous, and the differences between the calibrator, and the sample that presented the highest value of RQ was 0.46 (S4 Fig).

**Fig 3:**
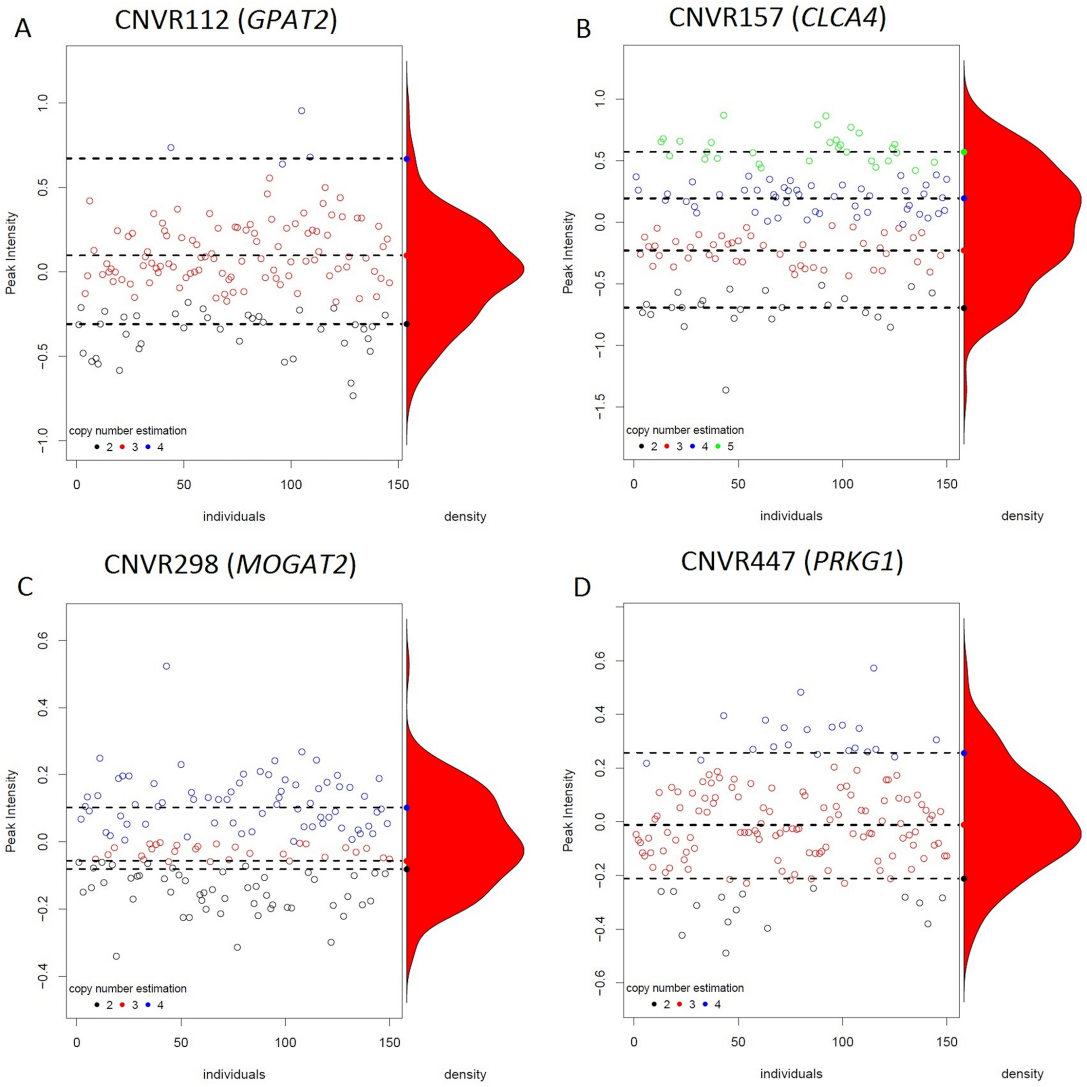
Plots of analyzed CNVRs generated from CNV signal-intensity data. The y-axis represents the CNV quantitative measurement (Peak Intensities) and the x-axis shows the different individuals. Each copy number estimation is shown in different colors. The density plot shows the distribution of these CNVs.

An association analysis between the CNV estimates of CNVR112, CNVR157, CNVR298, and CNVR447 and growth-related traits and FA composition in IMF and BF of BC1_LD animals was performed using CNVassoc R package [36]. The peak intensities (CNV quantitative measurement) and densities of the four analyzed CNVRs (CNVR112, CNVR157, CNVR298 and CNVR447) are shown in Fig 3. Four latent classes, corresponding to 2, 3, 4, and 5 copies for the CNVR157, were observed. For CNVR112, CNVR298 and CNVR447, three latent classes were observed corresponding to 2, 3 and 4 copies.

While for CNVR157, CNVR298 and CNVR447 only suggestive associations were found (S5 Table), CNVR112 (*GPAT2*) presented statistically significant associations (cut-off of q-value ≤ 0.05) for several traits. The strongest signal was observed for the C18:2(n-6)/C18:3(n-3) (p-value = 9.34E-05) ratio, and cis-vaccenic acid ((C18:1(n-7)) (p-value = 1.75E-03)) for the FA composition in BF. For FA percentages in IMF, the strongest signal was detected for oleic acid (C18:1(n-9) (p-value = 9.16E-04)), the global percentage of MUFA (p-value = 9.61E-04), peroxidability index (PI) (p-value = 3.70E-03), dihomo gamma linolenic acid (C20:3(n-6) (p-value = 5.51E-03)), the global percentage of PUFA (p-value = 6.21E-03), eicosatrienoic acid (C20:3(n-3) (p-value = 6.44E-03)) and the ratio of MUFA/PUFA (p-value = 9.18E-03). For growth traits, CRCL showed statistically significant association (p-value = 8.97E-05). These statistically significant associations and the descriptive statistics of significant phenotypic traits for CNVR112 are summarized in Table 3. A complete list of the association analyses results is shown in S5 Table, including the no significant associations observed in most of the FA composition traits measured in BF and IMF and for all the growth traits with the exception or CRCL.

**Table 3:** Statistically significant associations (q-value ≤ 0.05) between CNVR112 (*GPAT2*) and different phenotypic records in BC1_LD animals and their respective descriptive statistics.

Trait	N	Mean (SEM^a^)			p-value	q-value	Coefficient of determination^b^
**Composition of FA in BF**	147	**2 copies (n = 40)**	**3 copies (n = 103)**	**4 copies (n = 4)**			
C18:1(n-7)		0.13 (0.01)	0.14 (0.01)	0.14 (0.01)	1.75E-03	2.12E-02	0.12
C18:2(n-6)/C18:3(n-3)		19.27 (0.37)	18.29 (0.19)	17.06 (0.25)	9.34E-05	2.26E-03	0.17
**Composition of FA in IMF**	142	**2 copies (n = 37)**	**3 copies (n = 101)**	**4 copies (n = 4)**			
C18:1(n-9)		39.15 (0.49)	40.31 (0.27)	41.90 (0.81)	9.16E-04	1.01E-02	0.14
C18:2(n-6)		11.06 (0.43)	10.19 (0.23)	9.12 (0.38)	1.53E-02	2.41E-02	0.08
C20:2(n-6)		0.56 (0.02)	0.53 (0.01)	0.44 (0.03)	1.72E-02	2.41E-02	0.07
C20:3(n-3)		0.22 (0.02)	0.20 (0.02)	0.16 (0.02)	6.44E-03	2.26E-02	0.11
C20:3(n-6)		0.32 (0.02)	0.27 (0.01)	0.19 (0.04)	5.51E-03	2.26E-02	0.09
C20:4(n-6)		1.88 (0.15)	1.44 (0.06)	1.25 (0.18)	1.02E-02	2.28E-02	0.08
MUFA		46.97 (0.51)	48.18 (0.30)	49.89 (0.72)	9.61E-04	1.01E-02	0.13
PUFA		14.51 (0.62)	13.07 (0.31)	11.56 (0.56)	6.21E-03	2.26E-02	0.10
MUFA/PUFA		3.51 (0.19)	3.92 (0.10)	4.35 (0.24)	9.18E-03	2.28E-02	0.09
MUFA/SFA		1.23 (0.02)	1.25 (0.01)	1.30 (0.05)	1.21E-02	2.28E-02	0.08
PUFA/SFA		0.38 (0.02)	0.34 (0.01)	0.30 (0.02)	1.30E-02	2.28E-02	0.08
C18:1(n-9)/C18:0		2.78 (0.05)	2.85 (0.03)	3.05 (0.18)	1.05E-02	2.28E-02	0.09
C20:4(n-6)/C18:2(n-6)		0.16 (0.01)	0.14 (0.01)	0.14 (0.02)	1.65E-02	2.41E-02	0.07
PI		22.32 (1.09)	19.49 (0.49)	17.29 (1.14)	3.70E-03	2.26E-02	0.11
DBI		0.81 (0.01)	0.78 (0.01)	0.76 (0.02)	1.28E-02	2.28E-02	0.08
**Growth trait**	143	**2 copies (n = 36)**	**3 copies (n = 103)**	**4 copies (n = 4)**			
CRCL		86.4 (1.37)	83.53 (0.60)	81.00 (7.78)	8.97E-05	1.51E-03	0.17

^a^Standard error of the mean.

^b^The coefficient of determination reflects the genetic variability explained by CNVR112.

Interestingly, as stated before, CNVR112 contains the *GPAT2* gene, which plays a key role in phospholipid and triacylglycerol biosynthesis [53]. Triglycerides (TG) are the main constituents of body fat in higher eukaryotes, serving as the major energy storage [59]. Very low-density lipoproteins and chylomicrons derived from the liver and diet, respectively, are important sources of FA supply to several tissues such as the BF and muscle, determining their FA composition. Essential FAs provided by the diet may be directly stored or used to synthesize highly unsaturated FAs [60]. On the other hand, FA synthase releases palmitic acid (C16:0) from acetyl-CoA and malonyl-Coa which can be, in turn, the precursor of the long-chain saturated and unsaturated FAs of n-9 family (and minor FAs of the n-7 and n-10 families) [57]. Thus, CNVR112 may play a role in the genetic determination of IMF and BF FA composition traits through the synthesis of TG in BF and muscle, using FAs provided by diet or synthesized *de novo* in the liver or adipose tissue.

## Conclusions

This study is one of the first studies to investigate the association between CNVRs and economic traits in swine. We have described a map of swine CNVRs based on WGS data. A total of 540 CNVRs were identified across the autosomal chromosomes. Six selected CNVRs were validated by qPCR in three different backcrosses, and four of them were selected to study the association with FA composition in BF and IMF, and growth traits in 150 BC1_LD animals. CNVR112, which contains the *GPAT2* gene, showed associations with several of the analyzed growth-related traits and FA composition in IMF and BF.

These results indicate that CNVRs may explain a fraction of the genetic variability of FA composition, and also growth traits. These findings give novel insight into swine CNVRs and provide useful genomic information to facilitate the further identification of trait-related CNVRs.

## Supporting information

S1 Fig Analysis by qPCR of CNVR112 (*GPAT2*). The y-axis represents the RQ quantitative measurement by qPCR for each sample and the x-axis shows the different samples. The baseline represents the calibrator.
(TIFF)

S2 Fig Analysis by qPCR of CNVR157 (*CLCA4*). The y-axis represents the RQ quantitative measurement by qPCR for each sample and the x-axis shows the different samples. The baseline represents the calibrator.
(TIFF)

S3 Fig Analysis by qPCR of CNVR298 (*MOGAT2*). The y-axis represents the RQ quantitative measurement by qPCR for each sample and the x-axis shows the different samples. The baseline represents the calibrator.
(TIFF)

S4 Fig Analysis by qPCR of CNVR447 (*PRKG1*). The y-axis represents the RQ quantitative measurement by qPCR for each sample and the x-axis shows the different samples. The baseline represents the calibrator.
(TIFF)

S1 Table Primers used for qPCR assays. (XLSX)

S2 Table Duplication and deletion calls predicted by Control-FREEC software from all seven pigs. (XLSX)

S3 Table Information of 540 identified CNVRs and gene annotation within the CNVRs retrieved from the Ensembl Genes 84 Database using the Biomart data management system. (XLSX)

S4 Table Pathway analysis of genes identified in CNVRs. (XLSX)

S5 Table Association analysis between CNVRs and different phenotypic records in BC1_LD animals. (XLSX)
