# Supplementary figures and images for: A global analysis of CNVs in swine using whole genome sequence data and association analysis with fatty acid composition and growth traits
Source: PLoS One. 2017 May 4;12(5):e0177014. doi: 10.1371/journal.pone.0177014 (PMC5417718; doi:10.1371/journal.pone.0177014)

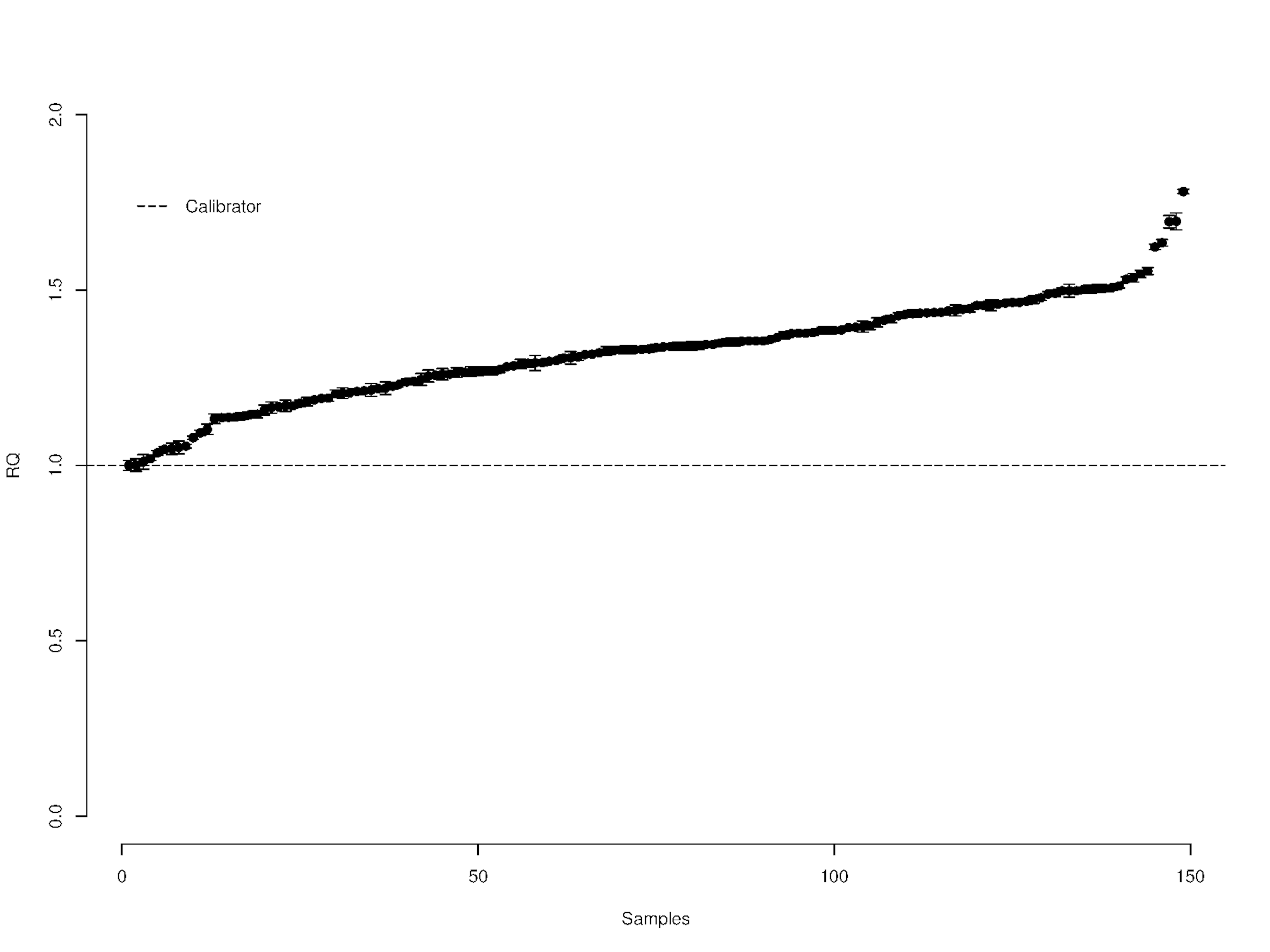

Supplement: S1 Fig — The y-axis represents the RQ quantitative measurement by qPCR for each sample and the x-axis shows the different samples. The baseline represents the calibrator. (TIFF) [file pone.0177014.s001.tiff]

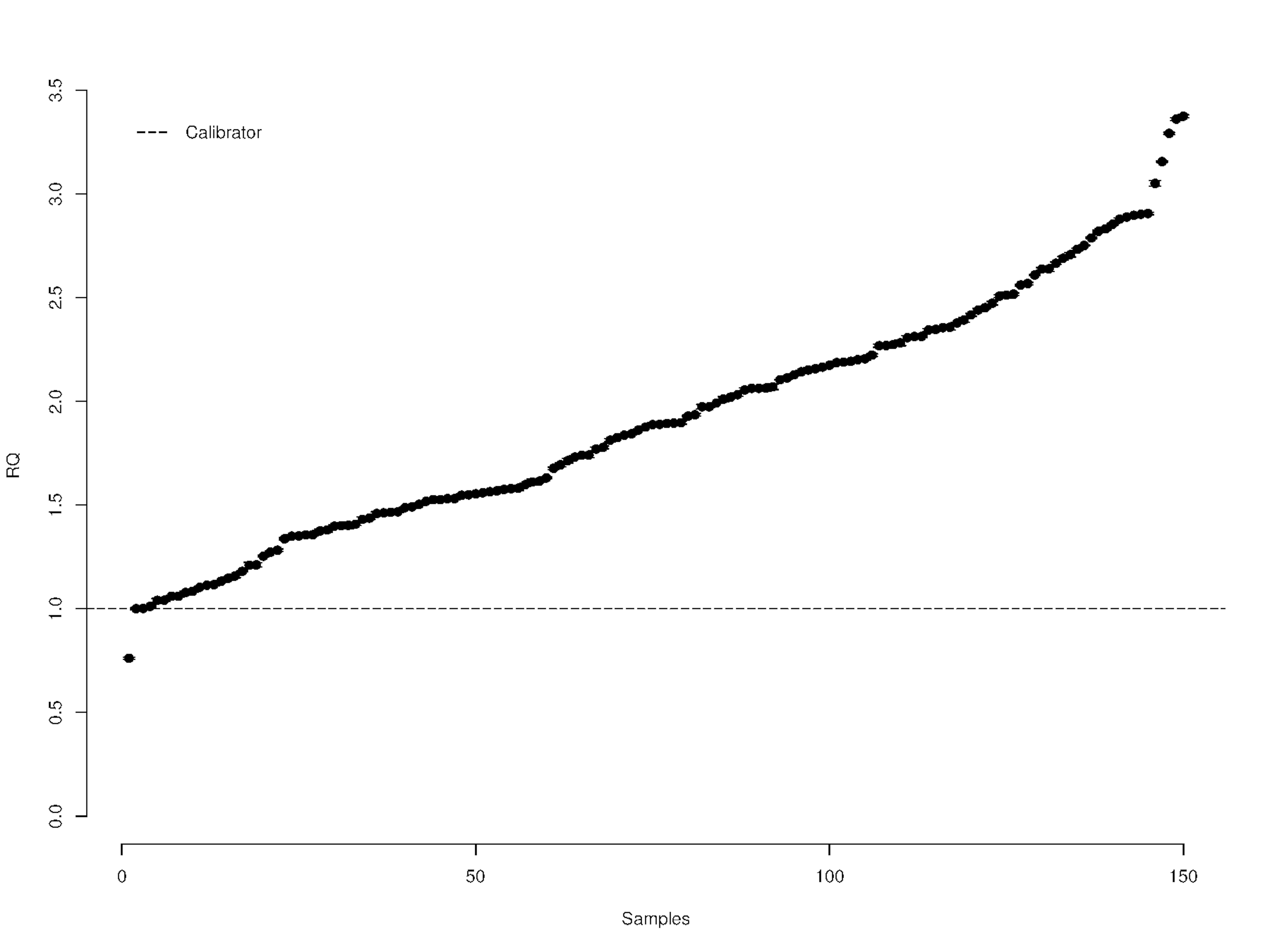

Supplement: S2 Fig — The y-axis represents the RQ quantitative measurement by qPCR for each sample and the x-axis shows the different samples. The baseline represents the calibrator. (TIFF) [file pone.0177014.s002.tiff]

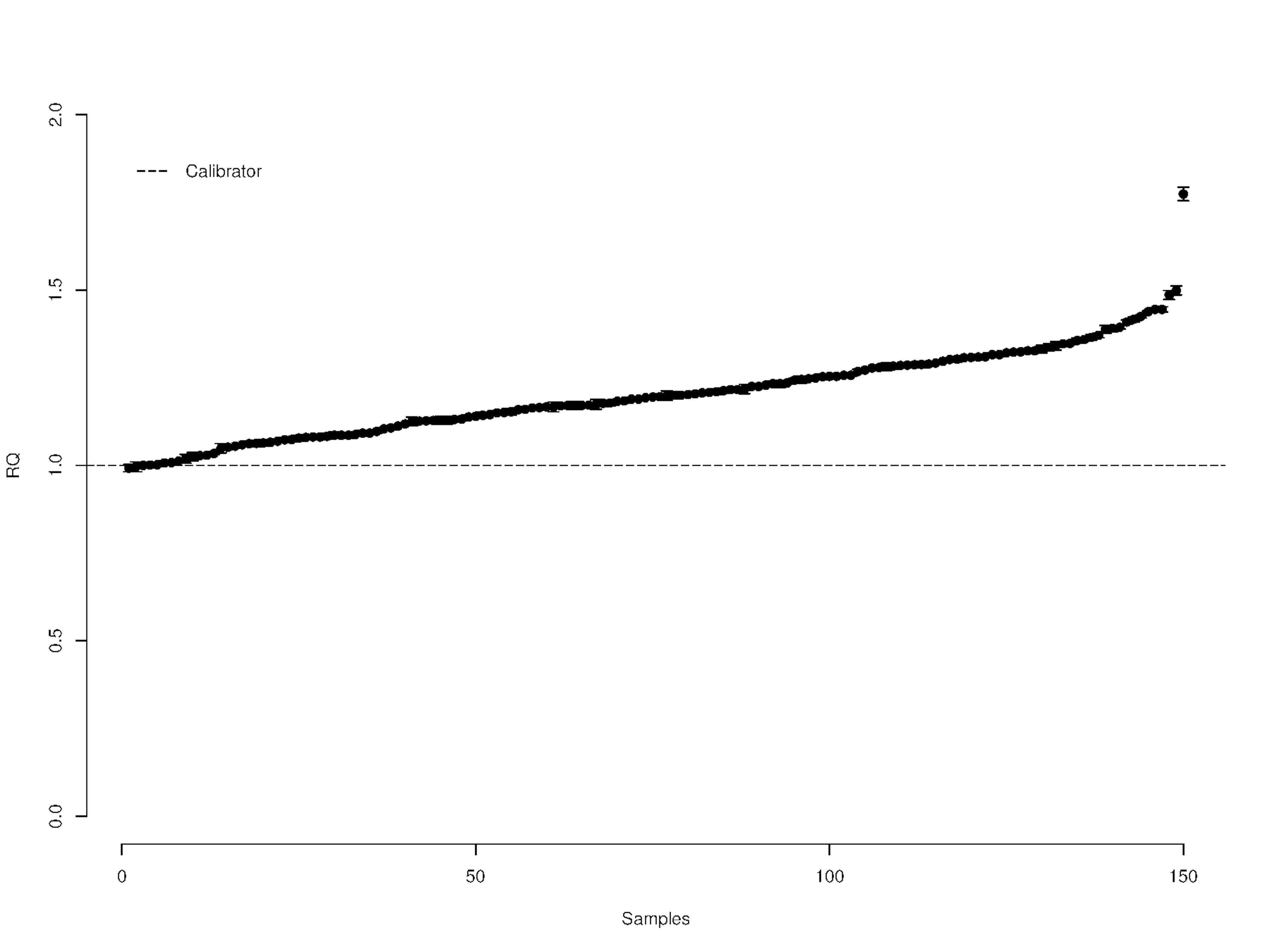

Supplement: S3 Fig — The y-axis represents the RQ quantitative measurement by qPCR for each sample and the x-axis shows the different samples. The baseline represents the calibrator. (TIFF) [file pone.0177014.s003.tiff]

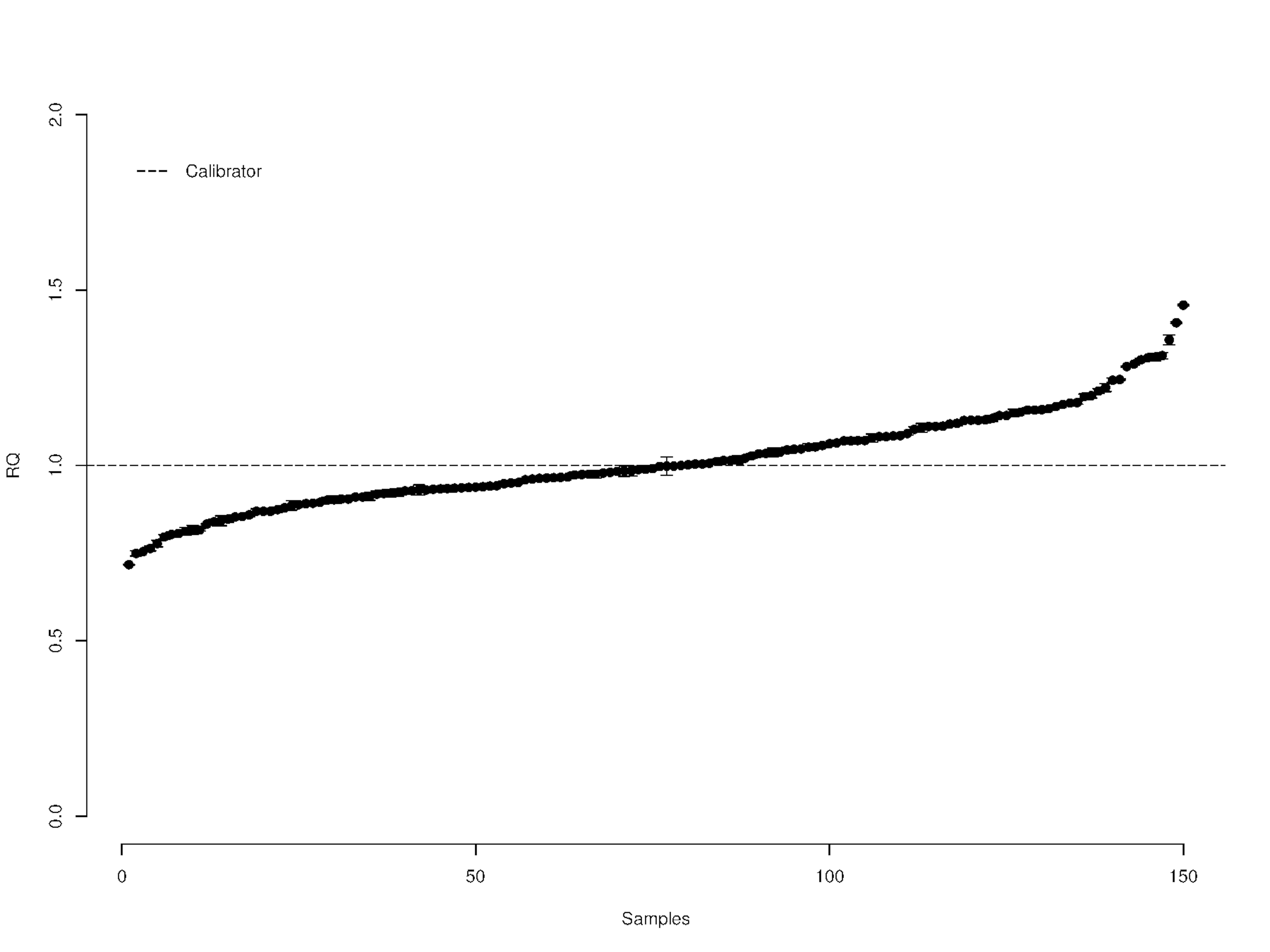

Supplement: S4 Fig — The y-axis represents the RQ quantitative measurement by qPCR for each sample and the x-axis shows the different samples. The baseline represents the calibrator. (TIFF) [file pone.0177014.s004.tiff]
